# Androgen deprivation therapy as adjuvant/neoadjuvant to radiotherapy for high-risk localised and locally advanced prostate cancer: recent developments

**DOI:** 10.1038/bjc.2011.385

**Published:** 2011-10-18

**Authors:** H Payne, M Mason

**Affiliations:** 1 Department of Clinical Oncology, University College Hospital, 235 Euston Road, London NW1 2BU, UK; 2 Department of Oncology and Palliative Medicine, Cardiff University Velindre Hospital, Whitchurch, Cardiff CF14 2TL, UK

**Keywords:** prostate cancer, androgen deprivation therapy, radiotherapy, neoadjuvant hormonal therapy, adjuvant hormonal therapy, GnRH antagonists

## Abstract

Androgen deprivation therapy (ADT) has traditionally formed the mainstay of treatment for advanced/metastatic prostate cancer (PCa); however, it is now also having an increasingly important role in earlier stages of disease. Indeed, in patients with locally advanced or high-risk localised disease, the addition of neoadjuvant and adjuvant hormone therapy is now considered the standard of care for those men treated with radical radiotherapy. Although luteinising hormone-releasing hormone (LHRH) agonists have been used for many years as ADT, they may be associated with clinical flare and testosterone breakthrough. Newer hormonal agents continue to be developed, such as gonadotropin-releasing hormone antagonists, which reduce testosterone and prostate-specific antigen levels more rapidly than LHRH agonists, without testosterone flare. This review examines ADT use in combination with radiotherapy to improve outcomes in localised or locally advanced disease, and examines some of the latest developments in hormonal therapy for PCa.

Androgen deprivation therapy (ADT) remains the mainstay of the medical management of advanced/metastatic prostate cancer (PCa) and is recommended as palliative treatment by current guidelines (Mottet *et al*, 2011; NCCN, 2011). ADT also has an increasingly important role in earlier stages of PCa, both as a monotherapy and combined with radical prostatectomy and radical radiotherapy. This review examines ADT use in combination with radiotherapy to improve outcomes in localised or locally advanced disease, as well as the latest developments in hormonal therapy for PCa.

## ADT in high-risk localised and locally advanced PCa

Management of locally advanced, non-metastatic (T3/T4) PCa presents two therapeutic challenges: the need for local control and the need to treat microscopic metastases undetectable with current imaging techniques. Therefore, a multimodal strategy should be considered. Treatment is, however, complicated by the absence of a universally agreed definition of ‘locally advanced’ disease amongst clinicians (oncologists and urologists; Payne and Gillatt, 2007). Furthermore, some localised tumours are also considered to be high risk. Thus, definitions of ‘high-risk’ PCa vary, but typically involve a combined assessment of tumour stage, Gleason grade and presenting prostate-specific antigen (PSA; see Tables 1 and 2). This review will refer to the high-risk group as men with locally advanced PCa (T3/T4) or localised disease with T1–T2 tumours, with Gleason grade⩾8 or a presenting PSA ⩾20 ng ml^−1^. The major modalities used in the treatment of high-risk localised and locally advanced PCa are summarised in Table 1.

### Radiotherapy alone

Treatment for locally advanced and high-risk localised PCa has traditionally included external beam radiotherapy (EBRT) alone, but despite improvements in radiotherapy techniques, many patients experience progression within 5 years (Shipley *et al*, 1999). A review of data from the pre-PSA era concluded that patients with locally advanced disease (stage C or T3) treated with EBRT had disease-free survival (DFS) rates of approximately 60–65% at 5 years, dropping to 30–40% at 10 years and 20–30% at 15 years (Aral *et al*, 2010). However, data (median 6.3 years’ follow-up) from the Mayo Clinic collaboration from patients with T1b–2 disease, who received EBRT from 1986 to 1995, and for whom PSA levels were available (Kuban *et al*, 2003) showed a PSA DFS rate of only 59% at 5 years and 53% at 8 years.

There is a wealth of strong evidence from multicentre, randomised clinical studies to support multimodality therapy with EBRT in combination with hormones. These studies demonstrated significant improvement in disease-specific and overall survival (OS) outcomes.

### Neoadjuvant hormonal therapy

Neoadjuvant hormonal therapy (NHT, i.e., therapy given before definitive local treatment with curative intent) aims to reduce tumour bulk and treat micrometastatic disease together with the primary lesion, although effects on metastases could be a consequence of local control rather than a systemic effect. Patients responding to NHT may also be candidates for adjuvant hormonal therapy after radiation, depending on pre-treatment risk factors.

NHT before definitive radiotherapy can lead, on average, to a 25–30% reduction in prostate size (Zelefsky *et al*, 1994; Henderson *et al*, 2003). The Radiation Therapy Oncology Group (RTOG) 86-10 study was the first Phase III trial to investigate the value of ADT in the neoadjuvant setting (Pilepich *et al*, 1995). Men with locally advanced PCa (large T2–4 tumours without bone metastases) received radiotherapy±combined androgen blockade (goserelin and flutamide), starting 2 months before and continuing during pelvic radiotherapy for a total duration of 4 months. In 456 evaluable patients (median follow-up 4.5 years), the 5-year incidence of local progression was significantly reduced with NHT (46%) *vs* radiotherapy alone (71% *P*<0.001). Similarly, the 5-year progression-free survival (PFS) with normal PSA levels was significantly greater with NHT (36%) than without (15% *P*<0.001), although there was no statistically significant decrease in the 5-year incidence of distant metastasis (34% *vs* 41%). Recent results (median follow-up 11.9–13.2 years) show that 10-year OS (43% *vs* 34%) and median survival (8.7 *vs* 7.3 years) were numerically superior in the NHT group, although the differences were not statistically significant (Roach *et al*, 2008). Disease-specific mortality (23% *vs* 36% *P*<0.01), distant metastasis (35% *vs* 47% *P*=0.006), DFS (11% *vs* 3% *P*<0.0001) and biochemical failure (PSA >2 ng ml^−1^; 65% *vs* 80% *P*<0.0001) were significantly superior in the NHT arm.

The Trans-Tasman Radiation Oncology Group (TROG) 96.01 trial compared radiotherapy alone with 3 or 6 months of NHT (goserelin and flutamide), given before (starting 2 and 5 months prior to radiotherapy, respectively) and during radiation, in men with locally advanced PCa (T2b-T4 N0 M0). The study included 802 evaluable patients and approximately 84% were high-risk (PSA >20 ng ml^−1^ or Gleason score >7 or stage T2c, T3, T4). Ten-year data showed that compared with radiotherapy alone, 3 and 6 months of NHT significantly reduced PSA progression (hazard ratio (HR)=0.72; *P*=0.003 and HR=0.57; *P*<0.0001, respectively) and local progression (HR=0.49; *P*=0.0005 and HR=0.45; *P*=0.0001, respectively), and improved event-free survival (HR=0.63; *P*<0.0001 and HR=0.51; *P*<0.0001, respectively; Denham *et al*, 2011). NHT for 6 months also significantly decreased distant progression (HR=0.49; *P*=0.001), PCa-specific mortality (HR=0.49; *P*=0.0008) and all-cause mortality (HR=0.63; *P*=0.0008), whereas 3-month NHT had no effect on these endpoints compared with radiotherapy alone. Although the TROG 96.01 trial provides long-term data on the treatment of men with locally advanced PCa with NHT, these results cannot be directly compared with those from RTOG 86-10 due to design differences – the RTOG study group received 4 months of NHT, with 2 months preceding radiotherapy.

### Adjuvant hormonal therapy

Emerging data from large, multi-centre, randomised trials demonstrated that early endocrine treatment of high-risk localised or locally advanced PCa after radical therapy can significantly delay disease progression and improve OS (e.g., Bolla *et al*, 1997; Messing *et al*, 1999; McLeod *et al*, 2006). Indeed, meta-analysis of seven studies of adjuvant hormonal therapy for locally advanced PCa showed that early intervention with ADT significantly reduces mortality and disease progression compared with late intervention (Boustead and Edwards, 2007).

#### Clinical trial data

Results from studies evaluating ADT adjuvant to radiotherapy are summarised in Table 2. The European Organisation for Research and Treatment of Cancer (EORTC) 22863 trial evaluated the effectiveness of 3 years of goserelin adjuvant to radiotherapy (Bolla *et al*, 2002). Patients with locally advanced, non-metastatic (T1–4, Nx, M0) PCa received radiotherapy with immediate goserelin (every 4 weeks for 3 years) or radiotherapy alone, with hormonal treatment after disease progression. After a median follow-up of 9.1 years, the 10-year OS and PFS rates were significantly higher for adjuvant goserelin *vs* radiotherapy alone (OS: HR=0.60, 95% CI: 0.45–0.80, *P*=0.0004; PFS: HR=0.42, 95% CI: 0.33–0.55, *P*<0.0001; Table 2; Bolla *et al*, 2009a).

RTOG 85-31 investigated long-term adjuvant goserelin in patients with locally advanced PCa and a high metastatic risk (T3 or regional lymphatic involvement), who had undergone definitive radiotherapy (Pilepich *et al*, 2005). The OS rate at 10 years was significantly greater for the adjuvant arm (49%) *vs* radiotherapy alone (39% *P*=0.002), with a significantly reduced risk of local failure or distant metastasis (Table 2). Notably, the greatest difference in OS was in men with Gleason score 8–10 (adjuvant arm, 39% radiotherapy alone, 25% *P*=0.0039). In RTOG 92-02, men with T2c–4 PCa received 4 months of ADT with goserelin and flutamide before and during radiotherapy, before being randomised to no additional therapy or 24 months of adjuvant goserelin (Hanks *et al*, 2003). Long-term adjuvant ADT was associated with significantly improved DFS, local progression, distant metastasis and biochemical failure, compared with short-term ADT, although there was no significant OS benefit in the full patient cohort. In a subset analysis in men with Gleason scores of 8–10, however, OS was significantly higher in the long-term adjuvant arm (81% *vs* 71% *P*=0.044; Hanks *et al*, 2003), supporting findings from RTOG 85-31. At long-term follow-up (median 11.3 years), benefits of adjuvant ADT on DFS, local progression and distant metastasis were maintained (Table 2) as was the benefit on OS in men with Gleason score 8–10 (45.1% *vs* 31.9% *P*=0.0061) although there was still no OS benefit in the full cohort (Horwitz *et al*, 2008).

Uncertainties remain regarding optimal timing and duration of hormone therapy, as timing has varied between trials. Goserelin was added during the final week of RTOG 85-31, the first week of EORTC 22863 and combined with 4 months of neoadjuvant and concomitant hormone therapy in RTOG 92-02. There were also differences in duration of adjuvant goserelin therapy between studies, with hormone therapy administered indefinitely (RTOG 85-31), for 3 years (EORTC 22863), or for 4 months in the neoadjuvant and 2 years in the adjuvant setting (RTOG 92-02). A recent study, using data from four institutions, suggested that the greatest benefits of adjuvant ADT occur after the first year, with relatively little benefit thereafter (Williams *et al*, 2011).

Quality of life is important when deciding treatment duration, and long-term side effects must be considered. The duration of adjuvant ADT (6 months *vs* 3 years) was investigated in EORTC 22961 (Bolla *et al*, 2009b). Men with locally advanced PCa (T1c–2b/N1–2/M0 or T2c–4/N0–2/M0) received 6 months of ADT with a luteinising hormone-releasing hormone (LHRH) agonist and were subsequently randomised to no additional ADT or a further 2.5 years of LHRH agonist treatment. Five-year overall mortality rates were numerically lower with long-term adjuvant ADT (Table 2; HR: 1.42; upper 95.71% confidence limit: 1.79). The authors concluded: ‘The combination of radiotherapy plus 6 months of androgen suppression provides inferior survival as compared with radiotherapy plus 3 years of androgen suppression in the treatment of locally advanced prostate cancer’.

Adjuvant therapy using the antiandrogen bicalutamide was evaluated in the Early Prostate Cancer Programme (McLeod *et al*, 2006). In locally advanced PCa (T3–4/M0 or N+/M0), bicalutamide adjuvant to radiotherapy significantly improved OS (70%) *vs* radiotherapy alone (58% HR=0.65; *P*=0.03) with a median follow-up of 7.4 years (Table 2). This was the first evidence of a significant OS benefit for any oral antiandrogen, given adjuvant to radical radiotherapy. Potential quality of life advantages have also been demonstrated with bicalutamide regarding potency, libido, physical capacity and preservation of bone mineral density at the cost of an increase in breast symptoms (Iversen *et al*, 2000; Sieber *et al*, 2004). The choice of treatment allows men to tailor side effects to their own needs and lifestyles.

Although the benefits of adding ADT to RT are now well established, Widmark *et al* (2009) examined whether ADT alone would give similar results in locally advanced PCa in the Scandinavian Prostate Cancer Group/Swedish Association for Urological Oncology (SPGC-7/SFUO-3) study. Men with locally advanced PCa (T1b–T2/G2–G3 or T3) and PSA ⩽70 ng ml^−1^ received complete androgen blockade with leuprolide and flutamide for 3 months, followed by radiotherapy or no additional treatment while continuing ADT with flutamide. At 10 years, addition of radiotherapy to ADT was associated with significantly reduced mortality (relative risk: 0.68; *P*=0.004; Table 2), and PSA recurrence at 10 years was significantly less frequent with ADT plus radiotherapy *vs* ADT alone (75% *vs* 26% relative risk: 0.16; *P*<0.0001; Widmark *et al*, 2009).

A similar design was used in the Intergroup PR3/PR07 study, which examined the effect on OS of radiotherapy added to lifelong ADT in 1205 men with locally advanced disease (T3–4/N0–X/M0; T2/N0–X/M0 + PSA >40 ng ml^−1^; or T2/N0–X/M0 + PSA >20 ng ml^−1^ + Gleason score ⩾8; Warde *et al*, 2010). Patients were randomised to lifelong ADT (bilateral orchidectomy or LHRH agonist)±radiotherapy. At median follow-up of 6.0 years, radiotherapy plus adjuvant ADT significantly reduced the overall mortality (HR: 0.77; *P*=0.033) and the disease-specific mortality (HR: 0.57; *P*=0.001) *vs* ADT alone. Grade ⩾2 late gastrointestinal toxicity rates were similar in both the arms.

This wealth of data from randomised, multicentre studies demonstrates that hormone therapy combined with radical radiotherapy is associated with significant benefits in local disease control, development of metastasis, DFS and OS. Combined modality treatment is now generally accepted as standard therapy for men with locally advanced or high-risk localised PCa, who are to be treated with radical intent. Present evidence supports 2–3 years of adjuvant ADT following radiotherapy (Bolla *et al*, 2009a), and recent data suggest the bulk of the benefit occurs in the first year (Williams *et al*, 2011). For NHT, therapy for 4 months (neoadjuvant for 2 months plus concomitant for 2 months) may be beneficial (Roach *et al*, 2008), but we await further results from ongoing studies to determine the optimal duration of NHT.

## New treatment options for ADT

Although LHRH agonists are highly effective in advanced PCa, they have several drawbacks including an initial testosterone surge (Sasagawa *et al*, 1998), which, in some patients, can lead to clinical flare (which may be potentially associated with skeletal pain, ureteral obstruction and spinal cord compression). Consequently, current guidelines recommend short-term use of an antiandrogen with LHRH agonist therapy (Mottet *et al*, 2011). Patients receiving LHRH agonists may also experience ‘testosterone breakthrough’ (i.e., a transient increase in testosterone above ‘castrate’ levels), which may have a negative impact on progression to castrate-resistant disease (Morote *et al*, 2007).

### GnRH antagonists

Gonadotropin-releasing hormone (GnRH) antagonists offer an alternative to LHRH agonists, with a more direct action as a result of immediate competitive binding to GnRH receptors (Van Poppel and Nilsson, 2008). One GnRH antagonist, abarelix, was initially licensed to treat advanced PCa (Weckermann and Harzmann, 2004), but is no longer marketed in the US. It is, however, still available in Germany.

**Figure 1:**
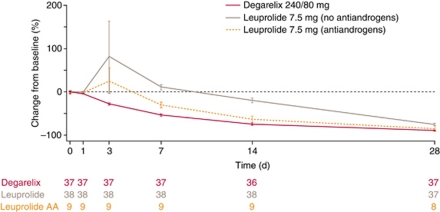
Percentage change from baseline in PSA (log-transformed mean (s.e.)) over the first 28 days in patients with metastatic disease at baseline, treated with leuprolide (with or without antiandrogens (AA)) or degarelix (Boccon-Gibod *et al*, 2011; reproduced with permission; Sage Publications)

Degarelix is a new GnRH receptor blocker/antagonist recently approved for hormone-dependent advanced PCa. In Phase II dose-finding studies, degarelix induced fast, profound and sustained testosterone suppression, with no evidence of testosterone surge (Gittelman *et al*, 2008; Van Poppel *et al*, 2008). Data from a 12-month, randomised, Phase III study in PCa patients (any stage) requiring ADT, showed that monthly degarelix had similar efficacy to leuprolide in terms of inducing and sustaining low testosterone levels (⩽0.5 ng ml^−1^) over the 1-year treatment period. Degarelix was not associated with testosterone surge or microsurges and achieved a more rapid suppression of luteinising hormone, testosterone and PSA than leuprolide, with evidence of better control of PSA (Figure 1; Klotz *et al*, 2008; Tombal *et al*, 2010; Boccon-Gibod *et al*, 2011). Risk of PSA progression or death was significantly lower with degarelix than with leuprolide (HR: 0.66; *P*<0.05; Tombal *et al*, 2010), and PSA progression occurred only in patients with baseline PSA >20 ng ml^−1^. In this subgroup, degarelix significantly extended the time to PSA recurrence *vs* leuprolide (*P*=0.04). Analysis of serum alkaline phosphatase (S-ALP), a marker of bone metabolism, has shown that patients with metastatic disease or PSA levels ⩾50 ng ml^−1^ at baseline in the Phase III study had greater reductions in S-ALP levels with degarelix compared with leuprolide, suggesting that degarelix might prolong control of skeletal metastases relative to leuprolide (Schröder *et al*, 2010).

Given the rapid onset of testosterone and PSA suppression with degarelix, there has been interest in the use of GnRH antagonists in combination with radiotherapy. In a study of NHT in 378 men with localised PCa, biochemical response (i.e., PSA reduction) to NHT was a more important predictor of therapeutic benefit than the duration of NHT (Alexander *et al*, 2010). Consequently, for patients who achieve a rapid fall in PSA after starting NHT, it may be possible to minimise the duration of ADT and its related toxicities. Thus, rapid biochemical control with GnRH antagonists may therefore shorten the duration of NHT. Preclinical data also suggest that tumour volume reduction may be greater with the blocker degarelix than with the agonist leuprolide (Princivalle *et al*, 2007). A comparative study of neoadjuvant degarelix or goserelin in men scheduled for radiotherapy is now underway (clinicaltrials.gov: NCT00833248).

### New hormonal agents for PCa

Promising results are now available with a number of new ADT therapies in the metastatic setting, of which the most notable are the cytochrome P450 17 (CYP17) inhibitors abiraterone and orteronel (TAK-700), and MDV-3100, a second-generation antiandrogen. However, the role of these therapies in earlier, high-risk disease remains to be determined.

Abiraterone is licensed for use in combination with prednisone for treatment of patients with metastatic castration-resistant PCa (mCRPC), who have received prior chemotherapy containing docetaxel; the application has been filed in the EU. Results from the pivotal randomised Phase III study (COU-AA-301) in this patient population (*n*=1195) demonstrated that abiraterone–prednisone significantly improved OS (14.8 *vs* 10.9 months, respectively; *P*<0.001), time to PSA progression (10.2 *vs* 6.6 months; *P*<0.001), radiographic PFS (5.6 *vs* 3.6 months; *P*<0.001) and PSA response rate (29% *vs* 6% *P*<0.001) compared with placebo-prednisone (de Bono *et al*, 2011). A randomised, double-blind, Phase III study in chemotherapy-naïve patients with mCRPC is ongoing and is scheduled to complete in April 2014 (clinicaltrials.gov: NCT00887198). In addition, an open-label, non-comparative Phase II study is investigating the combination of abiraterone and prednisone with conventional ADT before and during radiation therapy in patients with localised or locally advanced PCa (clinicaltrials.gov: NCT01023061). This trial is currently recruiting participants and results are expected in late 2014.

Orteronel is currently in Phase III development. Open-label Phase I data from 15 patients with mCRPC demonstrated that treatment with orteronel ⩾300 mg for three or more cycles was associated with PSA reductions ⩾50% in 12 patients (80%) and reductions ⩾90% in 4 patients (27% Dreicer *et al*, 2010). The Phase II portion of this study evaluating orteronel with concomitant prednisone is ongoing. Two randomised, double-blind, multicentre, Phase III clinical trials are currently recruiting patients with mCRPC. One study will evaluate orteronel plus prednisone compared with placebo plus prednisone in men with mCRPC that has progressed following taxane-based therapy (clinicaltrials.gov: NCT01193257), and the other study will compare these regimens in patients with chemotherapy-naïve mCRPC (clinicaltrials.gov: NCT01193244).

MDV3100 is also in Phase III development for the treatment of mCRPC. Long-term follow-up data from an open-label, non-comparative Phase I/II trial of 140 patients with mCRPC, who had received prior hormonal therapy (46% were chemotherapy-naïve and 54% had received previous chemotherapy), have shown that median time to PSA progression was 41 weeks for chemotherapy-naïve patients and 20 weeks for post-chemotherapy patients (Higano *et al*, 2011). Median time to radiological progression was 56 and 25 weeks, respectively. These results, along with an acceptable tolerability profile, have led to further clinical development of MDV3100 in two randomised, double-blind, placebo-controlled Phase III trials: AFFIRM (clinicaltrials.gov: NCT00974311) and PREVAIL (clinicaltrials.gov: NCT0121991). The AFFIRM trial will study the efficacy and safety of MDV3100 in patients with mCRPC previously treated with docetaxel-based chemotherapy, whereas PREVAIL is a safety and efficacy study of MDV3100 in chemotherapy-naïve patients with mCRPC. AFFIRM is ongoing and PREVAIL is currently recruiting participants.

Together, results from these novel hormonal agents show that men with ‘castrate-resistant’ PCa still maintain a degree of hormonal sensitivity and that further endocrine therapy after progression may be a viable option. On the basis of the utility of these agents in metastatic disease, future trials will help to clarify the optimal sequencing strategy and help clinicians identify the most suitable agent at each stage of the disease and for each patient population. Ideally, the early use of these agents in the sequence of therapies should not limit later choices of agents. However, data are not yet available to allow the discussion of potential positions for these agents in sequential regimens or whether they can be combined with other androgen axis-targeting agents with a different mechanism of action.

## Conclusion

Patients with locally advanced PCa may be at high risk of recurrence, metastases and PCa-related death. In these patients, as well as in those with high-risk localised disease, the addition of neoadjuvant and adjuvant hormone therapy is now considered a standard of care for those men treated with radical radiotherapy. Despite a period of 70 years since the importance of testosterone in the control of PCa cells was first described, there is still much interest in how to exploit this pathway for the treatment of PCa. The evidence base to determine optimal therapies and their timing is rapidly growing, and we await the results of trials of conventional combinations and newer targeted drugs with great interest.

LHRH agonists are effective for palliative treatment of advanced PCa, but may be associated with clinical flare and testosterone breakthrough. GnRH antagonists reduce testosterone and PSA levels more rapidly than LHRH agonists, and data from a Phase III comparative study show that the GnRH antagonist degarelix shows similar efficacy to leuprolide, with no testosterone flare, and evidence of better control of PSA and skeletal metastases. New hormonal agents in development have shown promising results in men with advanced, castrate-resistant PCa, and further data are eagerly awaited.

## Figures and Tables

**Table 1:** Major modalities of therapy for the primary treatment of high-risk localised and locally advanced prostate cancer (Heidenreich *et al*, 2011)

**Treatment**	**European Association of Urology recommendation for treatment**
Radical prostatectomy	Optional in selected patients with low-volume, high-risk localised PCa (cT3a or Gleason score 8–10 or PSA >20 ng ml^−1^)
	Optional in highly selected patients with very high-risk localised PCa (cT3b-T4 N0 or any T N1) in the context of multimodality treatment
Definitive radiotherapy	External irradiation with dose escalation is mandatory in high-risk patients (T2c or Gleason score >7 or PSA >20 ng ml^−1^)
	In daily practice, a combination of external irradiation with ADT is recommended (see below)
	In patients with locally advanced PCa (T3-4 N0 M0), combination with hormonal therapy is recommended (see below)
	Dose escalation may be of benefit in patients with bulky locally advanced PCa
Hormonal therapy (orchiectomy, diethylstilboestrol, LHRH agonists, degarelix)	Immediate castration for patients with locally advanced M0 PCa
	Non-steroidal antiandrogen monotherapy is an alternative to castration in patients with locally advanced PCa (T3-4, any N, or any T)
Combination therapy	Neoadjuvant hormonal treatment and concomitant hormonal therapy, plus radiotherapy in patients with high-risk localised PCa (T2c or Gleason score >7 or PSA >20 ng ml^−1^) improves survival
	In locally advanced PCa (T3-4 N0 M0), concomitant and adjuvant hormonal therapy (3 years) combined with external beam irradiation improves survival
	Patients <80 years with very high-risk PCa (c or pN1, M0) and no severe comorbidity may be candidates for external beam radiation therapy, plus immediate long-term hormonal manipulation

*Hormonal therapies in development* a	
Abiraterone: CYP17 inhibitor approved for use in combination with prednisone for the treatment of patients with mCRPC, who have received prior chemotherapy containing docetaxel	
Orteronel: CYP17 inhibitor in phase III development for the treatment of patients with mCRPC	
MDV3100: Second-generation antiandrogen in phase III development for the treatment of patients with mCRPC	

Abbreviations: ADT=androgen deprivation therapy; CYP17=cytochrome P450 17; mCRPC=metastatic castration-resistant prostate cancer; PCa=prostate cancer; PSA=prostate-specific antigen.

a At present, there is only one planned study of these agents (abiraterone) in combination with radiotherapy (source: clinicaltrials.gov).

**Table 2:** Studies evaluating ADT adjuvant to radiotherapy in men with high-risk prostate cancer

**Study**	**Population**	**Median follow-up (years)**	**ADT (randomisation groups)**	***N***	**Local failure (%)**	**Distant metastases (%)**	**Disease- free survival (%)**	**Overall survival (%)**
*5-Year estimates*								
EORTC 22863 (Bolla *et al*, 1997)	Locally advanced: T1–2/G3 or T3–4 N0-X M0	3.8	No additional therapy	198	8	48	48	62
			Goserelin	203	3	15	85^***^	79^**^
EORTC 22863 (Bolla *et al*, 2002)	Locally advanced: T1–2/G3 or T3–4 Nx M0	5.5	No additional therapy	198	16	29	40	62
			Goserelin	203	2^***^	10^***^	74^***^	78^***^
RTOG 92-02 (Hanks *et al*, 2003)	Locally advanced: T2c-4 N0 PSA <150 ng ml^−1^; 2 months of therapy with goserelin and flutamide before and 2 months during radiotherapy	5.8	No additional therapy	761	12	17	28	79
			Goserelin	753	6^***^	12^**^	46^***^	80
EORTC 22961 (Bolla *et al*, 2009b)	Locally advanced: T1c–T2b N1–2 M0 or T2c–T4 N0–2 M0 PSA ⩽40 × ULN; 6 months of therapy with CAB	6.4	No additional therapy	483	–	–	–	81
			LHRH agonist	487	–	–	–	85
								
*7-Year estimates*								
EPC (McLeod *et al*, 2006)	Locally advanced: T3–4 M0 or N+ M0	7.4	None	(1370)	–	–	–	58
			Bicalutamide		–	–	–	70^*^
SPCG-7/SFUO-3 (Widmark *et al*, 2009)	High-risk localised or locally advanced: T1b–T2/G2–G3 or T3 PSA ⩽70 ng ml^−1^	7.4–7.6	Flutamide	436	–	–	–	83
			Flutamide (no RT)	439	–	–	–	80
								
*10-Year estimates*								
RTOG 85-31 (Pilepich *et al*, 2005)	High-risk: T3 or regional lymphatic involvement	7.6	None	489	38	39	–	39
			Goserelin	488	23^**^	24^***^	–	49^**^
SPCG-7/SFUO-3 (Widmark *et al*, 2009)	High-risk localised or locally advanced: T1b–T2/G2–G3 or T3 PSA ⩽70 ng ml^−1^	7.6	Flutamide	436	–	–	–	70^**^
			Flutamide (no RT)	439	–	–	–	61
EORTC 22863 (Bolla *et al*, 2009a)	Locally advanced: T1–2/G3 M0 or T3–4 N0–1 M0	9.1	No additional therapy	198	–	70	23	40
			Goserelin	203	–	49^***^	48^***^	58^***^
RTOG 92-02 (Horwitz *et al*, 2008)	Locally advanced: T2c-4 N0–X PSA <150 ng ml^−1^; 4 months of therapy with goserelin and flutamide before and during radiotherapy	11.3	No additional therapy	763	22.2	22.8	13.2	51.6
			Goserelin (24 months)	758	12.3^***^	14.8^***^	22.5^***^	53.9

Abbreviations: ADT=androgen deprivation therapy; EPC=Early Prostate Cancer; EORTC=European Organisation for Research and Treatment of Cancer; LHRH=luteinising hormone-releasing hormone; PCa=prostate cancer; PSA=prostate-specific antigen; RT=radiotherapy; RTOG=Radiation Therapy Oncology Group; SPGC-7/SFUO-3=Scandinavian Prostate Cancer Group/Swedish Association for Urological Oncology; ULN=upper limit of normal.

^*^P<0.05; ^**^P<0.01; ^***^P<0.001 *vs* control arm.
